# *CYP7A1* Gene Polymorphism Located in the 5′ Upstream Region Modifies the Risk of Coronary Artery Disease

**DOI:** 10.1155/2015/185969

**Published:** 2015-04-06

**Authors:** Tomasz Iwanicki, Anna Balcerzyk, Pawel Niemiec, Tomasz Nowak, Anna Ochalska-Tyka, Jolanta Krauze, Sylwia Kosiorz-Gorczynska, Wladyslaw Grzeszczak, Iwona Zak

**Affiliations:** ^1^School of Health Sciences in Katowice, Medical University of Silesia, Department of Biochemistry and Medical Genetics, Medykow Street 18, 40-752 Katowice, Poland; ^2^Regional Centre of Blood Donation and Blood Treatment in Raciborz, Sienkiewicza Street 3, 47-400 Raciborz, Poland; ^3^School of Medicine in Katowice, Medical University of Silesia, 1st Department of Cardiac Surgery in Upper Silesian Center of Cardiology in Katowice, Ziolowa Street 47, 40-635 Katowice, Poland; ^4^School of Medicine with the Division of Dentistry in Zabrze, Medical University of Silesia, Department of Internal Medicine, Diabetes and Nephrology, 3 Maja Street 13-18, 41-800 Zabrze, Poland

## Abstract

*Background*. 7-Alpha cholesterol hydroxylase (CYP7A1), the first enzyme of classic conversion pathway leading from cholesterol to bile acids synthesis, is encoded by *CYP7A1* gene. Its single nucleotide polymorphisms (SNPs) influence serum lipid levels and may be related to impaired lipid profile leading to coronary artery disease (CAD). The aim of the present study was to analyze the possible association between the rs7833904 *CYP7A1* polymorphism and premature CAD. *Material and Methods*. Serum lipid levels and rs7833904 SNP were determined in 419 subjects: 200 patients with premature CAD and 219 age and sex matched controls. *Results*. The A allele carrier state was associated with CAD (OR = 1.76, 95% CI; 1.14–2.71, *P* = 0.014). The effect was even stronger in the male subgroups (OR = 2.16, 95% CI; 1.28–3.65, *P* = 0.003). There was no effect in the females. Risk factors of CAD and clinical phenotype of atherosclerosis were not associated with genotype variants of the rs7833904 SNP. Lipid profiles also did not differ significantly between individual genotypes. *Conclusion*. The *CYP7A1* rs7833904 polymorphism may modify the risk of CAD. This effect is especially strong in male subjects. The studied polymorphism does not significantly influence serum lipid levels, in the present study.

## 1. Introduction

Coronary artery disease (CAD) is the clinical consequence of atherosclerotic plaque formation. CAD is classified as an inflammatory and multifactorial disease [1], because of numerous genetic and environmental factors involved in the development of atherosclerosis. One of the most important risk factors of CAD is impaired lipid profile.

7-Alpha cholesterol hydroxylase (CYP7A1) is the first enzyme of classic conversion pathway leading from cholesterol to bile acids synthesis, which is the main way of cholesterol removal from the body [2]. In contrast to the alternative pathway, it is regulated by the negative feedback mechanism [3]. CYP7A1 belongs to the large family of the p450 cytochrome proteins.* CYP7A1* gene encoding CYP7A1 enzyme is located on the 8q11-12 chromosome and it consists of 6 exons and 5 introns [4]. Among a large number of polymorphic variants in the* CYP7A1* gene, Nakamoto et al. [5] typed six haplotype-tagging single nucleotide polymorphisms (htSNPs) for Caucasian population.

Due to the role of the CYP7A1 as the key element of cholesterol conversion, the* CYP7A1* gene polymorphisms were examined for its potential effects on lipid metabolism. It was shown that the* CYP7A1* genetic variants influenced serum levels of LDL lipoproteins and triacylglycerols [6–8]. A vast majority of these studies concerned the rs3808607 SNP (−278A/C), the polymorphism located in the promoter region of the gene. Moreover, a few of these studies reported the associations between the* CYP7A1* gene polymorphisms and such diseases like gallbladder stone [9], gallbladder cancer [10, 11], proximal colon cancer [12–15],* neuromyelitis optica* [16], or coronary artery disease [8, 17, 18].

There are also some polymorphisms of the* CYP7A1* gene which were not studied in a context of CAD. Therefore the aim of the present study was to analyze the possible association between the rs7833904 polymorphism and premature coronary artery disease. The rs7833904 polymorphism is in the complete linkage disequilibrium with rs1023652, in which genotypes differentiate the concentration of the hyodeoxycholic bile acid [19]. These two polymorphisms are representative for the haplotype block 2 in Caucasians [5].

We also examined whether genetic variants of the rs7833904 SNP affect plasma lipid concentrations. This study may help in better understanding of genetic background of CAD in Polish population, which is at high risk of the disease, what may be partly explained by the specific genetic background.

## 2. Materials and Methods

### 2.1. Study Subjects

We studied 419 subjects, inhabitants of Upper Silesia. Only white Polish Caucasians were included in the study. First group (CAD) consisted of 200 patients with angiographically proven premature CAD (62 females and 138 males), aged 28–55 years (mean 43.49 ± 6.16). Second, control group included 219 blood donors (63 females and 156 males), with no signs of CAD and negative familial history of the disease. Age mean of this group was 44.64 ± 6.33. CAD subjects were selected from patients admitted to the (1) 1st Department and Clinic of Cardiology at the Upper Silesian Centre of Cardiology in Katowice and (2) 1st Department of Cardiac Surgery at the Upper Silesian Centre of Cardiology in Katowice.

They were classified for the study by the same cardiologist. Controls were recruited from the Regional Centers of Blood Donation and Blood Treatment in Katowice and Raciborz. Blood samples were gathered only from subjects with systolic blood pressure (BP) < 140 and diastolic BP < 90 on the day of blood collection in accordance with recommendations of the Polish Centers of Blood Donation and Blood Treatment.

Inclusion and exclusion criteria, details of the medical interview, and diagnosis and evaluation as well as criteria for CAD, myocardial infarction (MI), and traditional risk factors of CAD were described previously [20].

The study protocol was approved by the Ethics Committee of the Medical University of Silesia in Katowice, Poland. All subjects gave written informed consents.

### 2.2. Biochemical Analyses

Total serum cholesterol (TC), HDL cholesterol (HDL-C), and triacylglycerols (TG) were measured by enzymatic methods (Analco, Warsaw, PL). LDL cholesterol (LDL-C) levels were calculated according to the Friedewald formula [21] in subjects with triacylglycerols levels below 4.4 mmol/L.

### 2.3. Genetic Analyses

Genomic DNA was isolated from leukocytes of peripheral blood using the MasterPure genomic DNA purification kit (Epicentre Technologies, Madison, USA). The rs7833904 polymorphism of the* CYP7A1* gene was genotyped using the TaqMan Predesigned SNP Genotyping Assay (Applied Biosystems, Foster City, USA).

Total volume of 20 *μ*L of reaction mix included 10 *μ*L of TaqMan Genotyping Master Mix (Cat.# 4371355), 1 *μ*L of probe (TaqMan Predesigned SNP Genotyping Assay, Cat.# 4351379; ID C_11309045_10), 1 *μ*L of DNA template (15 ng/*μ*L), and 8 *μ*L of deionized water. Probe was diluted with the TE buffer (1 : 1) before the reaction. Polymerase chain reaction amplification was performed according to the manufacturer's specifications. Genotyping was performed using the 7300 Real-Time PCR System (Applied Biosystems). 30% of samples were regenotyped to exclude the genotyping errors. Repeatability of results was 100%.

### 2.4. Statistical Analysis

Data were analyzed using* Statistica 10.0* (STATSOFT, Tulsa, OK, USA) and SNPator [22] software.

Normality of distribution was assessed by the Shapiro-Wilk test and then a comparison of quantitative data was performed by the Mann-Whitney *U* test (for variables with nonnormal distribution) or Student's *t*-test (for variables with normal distribution). Allele frequencies were calculated from the genotypes distributions. Hardy-Weinberg equilibrium was tested in all groups by a *χ* ^2^ test as well as comparisons of genotypes and alleles frequencies between cases and controls. Fisher's correction was used when the number of subjects in the specific subgroup was below 10. Odds ratios (OR) with their 95% confidence intervals (CI) were computed using a univariate analysis and multiple logistic regression analysis after adjustment for traditional risk factors of CAD (cigarette smoking, serum lipid levels of TC, LDL-C, HDL-C and TG, BMI, hypertension, and diabetes mellitus). The effective sample size and statistical power of association analyzes were computed using* Epi Info 7.1.1.0* developed by Centers for Disease Control and Prevention.

## 3. Results

Characteristics of the patients from the studied groups are shown in Table 1. The occurrence of myocardial infarction in the CAD group was 75% (*n* = 150). 61% of patients (*n* = 123) had critical stenosis in the coronary vessels; 97% of patients (*n* = 194) were treated with statins. There is no information about statins therapy in the blood donors group, but taking into account the inclusion criteria (no symptoms of CAD and its negative family history), it is highly doubtful that these subjects were treated with statins. CAD group differed significantly from controls in such parameters as BMI, TC, LDL-C, HDL-C, and TG levels.

Genotypes frequencies of both studied groups were compatible with Hardy-Weinberg equilibrium (*P* = 0.86 for the CAD group; *P* = 0.38 for the control group). Genotyping results are shown in Table 2. We found statistically significant differences in the frequencies of genotypes and alleles of the rs7833904 polymorphism between patients and controls. The A allele carrier state was more frequent in the CAD group in comparison to the controls (77.5% versus 66.21%) and this difference was statistically significant (*P* = 0.014). The power of the test was 72%, with a 95% two-sided confidence level. The frequency of the A allele in the CAD group was also significantly higher than in the control group (*P* = 0.005). The power of this comparison was 80% with 95% CI. Similar and ever stronger effects were shown in the subgroups of males (Table 3). The A allele carrier state was more frequent in the male patients than in the healthy males (78.98% versus 63.46%, *P* = 0.005). The power of the test was 94% (95% CI). The frequency of A allele also highly differentiated male subgroups (55.07% versus 40.70%, for CAD patients and blood donors, resp., *P* = 0.0006). The power of this test was 84.5% (95% CI). These differences were not observed in the female subgroups (Table 4).

The rs7833904 genotypes and alleles were tested for interactions with such risk factors of atherosclerosis as hypertension, diabetes mellitus, BMI, and cigarette smoking but without any notable effect (data not shown). Parameters of clinical CAD phenotype like critical stenosis of coronary arteries, multivessel stenosis, MI, or left ventricular hypertrophy also were not associated with genotype variants of the rs7833904 SNP (data not shown).

TC, HDL-C, LDL-C, and TG levels were compared between particular genotypes of the rs7833904 polymorphism (Table 5). Lipid profiles did not differ significantly between the genotypes, except of the levels of triacylglycerols. TG levels increased in relation to the T allele dose (Table 4). This trend was observed only in the blood donors group; however, the differences between respective genotypes lied close to the threshold of statistical significance (AA versus TT, *P* = 0.06; AT versus TT, *P* = 0.08; AA versus AT, *P* = 0.42).

## 4. Discussion

The results of our study showed that carriers of the A allele were more frequent in the CAD group than in the control group. It suggests an association between the A allele and increased risk of coronary artery disease. In contrast, higher frequency of the TT genotype was characteristic for the control group and this genotype seems to be genetic preventive factor from CAD. This relationship was also confirmed in the male subgroups but was not observed in women. The association between the rs7833904 polymorphism of the* CYP7A1* gene and coronary artery disease was described for the first time. The potential role of the rs7833904 polymorphism in the development of CAD was not previously analyzed. The functional significance of the rs7833904 polymorphism is also not known, but another* CYP7A1* gene polymorphism (rs1023652) is in a complete linkage disequilibrium with the rs7833904. The A allele of the rs7833904 is inherited together with G allele of the rs1023652 [5]. Xiang et al. [19] showed that the GG homozygotes of the rs1023652 had significantly lower concentration of the hyodeoxycholic bile acid (HDCA) than subjects with CG or CC genotype (−41% and −34%, resp.). Thus, it is possible that the polymorphism analyzed in our study modifies the risk of CAD development by influence on bile acids concentration. The effect of bile acid disposal on coronary artery atherosclerosis was examined by Charach et al. [23]. The study showed that CAD patients had much lower deoxycholic acid, lithocholic acid, and total amount of bile acids in comparison to controls. The authors supposed that lower bile acids concentrations might be caused by the reduced CYP7A1 activity or impaired ability of the body to increase the concentration of this enzyme. It is reasonable to speculate that the A allele of the rs7833904 polymorphism reduces the ability of the enzyme to convert cholesterol to bile acids, which may lead to increase in the cholesterol level, with the subsequent development of atherosclerosis. The studies on laboratory animals confirm an association between bile acids amount and cholesterol level. Supplementation of hamster's diet with hyodeoxycholic acid caused 21% decrease in the total cholesterol concentration and significant lowering of LDL and VDL plasma concentration [24]. Our further analysis, however, did not reveal any association between the rs7833904 polymorphism and total cholesterol, LDL and HDL concentrations. We also did not find any associations between genotypes and alleles of the studied polymorphism and other traditional risk factors of atherosclerosis.

It should be noted that nearly all patients were treated with statins in our study, which might influence the results of the analysis of the association between the studied genetic variant and cholesterol level in CAD group.* CYP7A1* gene polymorphisms may also modify response of organism to lipid lowering drugs [25, 26], which may be another reason of the obtained results. Nevertheless, it is difficult to explain the lack of association between the rs7833904 polymorphism and cholesterol level in the control group; however, our findings are in line with Xiang et al. [19] who observed an association of rs1023652 polymorphism (being with complete linkage disequilibrium with the rs7833904) with HDCA amount, but not with the concentrations of total cholesterol and 7*α*-hydroxy-4-cholesten-3-one (7HCO, the known marker of the primary bile acids synthesis). Charach et al. [23] also did not observe an association between bile acids secretion and TC and LDL concentration but he reported a positive correlation between plasma triacylglycerols and bile acid excretion in the non-CAD group. He supposed that CAD patients did not exhibit this effect because the amount of excreted bile acids was significantly lower in this group. In our control group we observed a tendency to higher concentration of triacylglycerols in subjects with TT genotype comparing to AA homozygotes, where the difference was close to the threshold of significance (*P* = 0.06). Assuming that the A allele is associated with lower bile acids amount, TT homozygotes would have higher level of bile acids which might cause rapid and more complete intestinal absorption of triacylglycerols due to an excess of bile acids which are necessary for the emulsification of fats.

## 5. Conclusions

In conclusion, the* CYP7A1* rs7833904 polymorphism may modify the risk of CAD. This effect is especially strong in male subjects. The studied polymorphism does not significantly influence serum lipid levels, in the present study. Further studies, including bile acids concentration, lipid parameters, and CYP7A1 activity are necessary to explain the role of the* CYP7A1* gene and its genetic variants in CAD pathology.

## Figures and Tables

**Table 1:** Traditional risk factors of CAD in the group of coronary artery disease (CAD) patients and controls.

Group	CAD *N* = 200	Control *N* = 219
Male sex, *n* (%)	138 (69.00)	156 (71.23)
Age (years), mean ± SD	43.49 ± 6.16	44.64 ± 6.33
BMI, mean ± SD	26.94 ± 4.06^*^	25.90 ± 3.77
TC (mmol/L), mean ± SD	5.86 ± 1.38^*^	5.11 ± 1.21
LDL-C (mmol/L), mean ± SD	4.07 ± 1.24^*^	3.15 ± 1.16
HDL-C (mmol/L), mean ± SD	1.04 ± 0.30^*^	1.45 ± 0.57
TG (mmol/L), mean ± SD	1.93 ± 1.03^*^	1.40 ± 0.72
Smoking, *n* (%)	58 (26.36)^*^	123 (61.19)

^*^ *P* < 0.05—statistically significant differences between groups.

**Table 2:** Distribution of the genotypes and alleles of the rs7833904 polymorphism in the group of coronary artery disease (CAD) patients and controls.

Genotype/allele	CAD *N* = 200	Control *N* = 219	Model	OR (CI 95%), *P*
	*n* (%)	*n* (%)		
TT	45 (22.50%)	74 (33.78%)	Additive	1
AT	98 (49.00%)	101 (46.12%)		1.59 (1.00–2.53) 0.06
AA	57 (28.50%)	44 (20.10%)		2.13 (1.24–3.66), 0.008^*^
AA + AT TT	155 (77.50%) 45 (22.50%)	145 (66.21%) 74 (33.78%)	Dominant	1.76 (1.14–2.71), 0.014^*^ 1
AT + TT AA	143 (71.50%) 57 (28.50%)	175 (79.90%) 44 (20.10%)	Recessive	0.63 (0.40–0.99), 0.058 1

A	212 (53.00%)	189 (43.15%)		1.49 (1.13–1.95), 0.005^∗ vs T^
T	188 (47.00%)	249 (56.85%)		0.67 (0.51–0.88), 0.005^∗ vs A^

^*^ *P* < 0.05—statistically significant differences between groups.

**Table 3:** Distribution of the genotypes and alleles of the rs7833904 polymorphism in the male subgroups of coronary artery disease (CAD) patients and controls.

Genotype/allele	CAD *N* = 138	Control *N* = 156	Model	OR (CI 95%), *P*
	*n* (%)	*n* (%)		
TT	29 (21.02%)	57 (36.54%)	Additive	1
AT	66 (47.82%)	71 (45.51%)		1.82 (1.04–3.19), 0.047^*^
AA	43 (31.16%)	28 (17.95%)		3.01 (1.57–5.79), 0.001^*^
AA + AT TT	109 (78.98%) 29 (21.02%)	99 (63.46%) 57 (36.54%)	Dominant	2.16 (1.28–3.65), 0.005^*^ 1
AT + TT AA	95 (68.84%) 43 (31.16%)	128 (82.05%) 28 (17.95%)	Recessive	0.48 (0.28–0.83), 0.012^*^ 1

A	152 (55.07%)	127 (40.70%)		1.79 (1.28–2.48), 0.0006^∗ vs T^
T	124 (44.93%)	185 (59.3%)		0.56 (0.40–0.77), 0.0006^∗ vs A^

^*^ *P* < 0.05—statistically significant differences between groups.

**Table 4:** Distribution of the genotypes and alleles of the rs7833904 polymorphism in the female subgroups of coronary artery disease (CAD) patients and controls.

Genotype/allele	CAD *N* = 62	Control *N* = 63	Model	OR (CI 95%), *P *
	*n* (%)	*n* (%)		
TT	16 (35.56%)	17 (22.97%)	Additive	1
AT	32 (32.65%)	30 (29.70%)		1.70 (0.70–4.11), 0.338
AA	14 (24.56%)	16 (36.26%)		0.93 (0.34–2.50), 0.914
AA + AT TT	46 (74.19%) 16 (35.56%)	46 (73.01%) 17 (22.97%)	Dominant	1.06 (0.47–2.38), 0.881 1
AT + TT AA	48 (77.41%) 14 (24.56%)	47 (74.60%) 16 (36.26%)	Recessive	1.16 (0.51–2.70), 0.712 1

A	60 (48.38%)	62 (49.20%)		1.03 (0.63–1.70), 0.897^vs T^
T	64 (51.62%)	64 (50.80%)		0.97 (0.58–1.59), 0.897^vs A^

**Table 5:** Serum lipid levels and BMI values with respect to genotypes and alleles of the rs7833904 polymorphism in the groups of coronary artery disease (CAD) patients and controls.

Control					
Genotype/allele	TC mmol/L ± SD	LDL-C mmol/L ± SD	HDL-C mmol/L ± SD	TG mmol/L ± SD	BMI kg/m^2^ ± SD
AA	4.95 ± 1.02	2.92 ± 0.92	1.56 ± 0.61	1.25 ± 0.60	25.40 ± 3.83
AT	5.18 ± 1.27	3.23 ± 1.23	1.46 ± 0.58	1.34 ± 0.61	25.91 ± 3.82
TT	5.12 ± 1.19	3.21 ± 1.08	1.35 ± 0.46	1.54 ± 0.80	26.29 ± 3.43
A	5.11 ± 1.20	3.13 ± 1.70	1.49 ± 0.59	1.31 ± 0.60	25.76 ± 3.81
T	4.95 ± 1.02	2.92 ± 0.98	1.56 ± 0.61	1.25 ± 0.60	25.41 ± 3.83

CAD					
Genotype/allele	TC mmol/L ± SD	LDL-C mmol/L ± SD	HDL-C mmol/L ± SD	TG mmol/L ± SD	BMI kg/m^2^ ± SD

AA	5.76 ± 1.30	3.99 ± 1.16	1.10 ± 0.31	2.00 ± 1.05	27.08 ± 4.22
AT	5.80 ± 1.36	3.97 ± 1.22	1.13 ± 0.31	1.97 ± 1.11	26.75 ± 4.04
TT	5.94 ± 1.50	4.20 ± 1.13	1.12 ± 0.31	1.80 ± 0.85	27.45 ± 3.67
A	5.79 ± 1.33	3.98 ± 1.20	1.12 ± 0.30	1.98 ± 1.08	26.87 ± 4.10
T	5.85 ± 1.42	4.04 ± 1.27	1.12 ± 0.30	1.92 ± 1.03	26.98 ± 3.92
